# Visible-light optical coherence tomography: a review

**DOI:** 10.1117/1.JBO.22.12.121707

**Published:** 2017-12-07

**Authors:** Xiao Shu, Lisa Beckmann, Hao F. Zhang

**Affiliations:** a Northwestern University, Department of Biomedical Engineering, Evanston, Illinois, United States; b Northwestern University, Department of Ophthalmology, Chicago, Illinois, United States

**Keywords:** optical coherence tomography, retinal imaging, metabolic rate of oxygen, hemodynamics, brain imaging, spectroscopic analysis

## Abstract

Visible-light optical coherence tomography (vis-OCT) is an emerging imaging modality, providing new capabilities in both anatomical and functional imaging of biological tissue. It relies on visible light illumination, whereas most commercial and investigational OCTs use near-infrared light. As a result, vis-OCT requires different considerations in engineering design and implementation but brings unique potential benefits to both fundamental research and clinical care of several diseases. Here, we intend to provide a summary of the development of vis-OCT and its demonstrated applications. We also provide perspectives on future technology improvement and applications.

## 1 Introduction

First reported in the 1990s,^1^^–^^3^ optical coherence tomography (OCT) has been one of the most successful technologies to exhibit rapid clinical translation and adoption. It provides three-dimensional (3-D) *in vivo* optical tissue biopsy noninvasively with fine resolution in both lateral and axial dimensions at a penetration depth up to a few millimeters.^4^ OCT has been applied in various investigational and clinical fields, including dermatology,^5^ cardiology,^6^ urology,^7^ dentistry,^8^ oncology,^9^ pulmolory,^10^ and, most prominently, ophthalmology.^11^ In addition to 3-D structural imaging, extensions of OCT technology can provide functional information of biological tissue.^12^ Birefringence properties, flow velocity, and molecular contrast can be obtained by polarization-sensitive OCT,^13^ Doppler OCT,^14^ and spectroscopic OCT (sOCT),^15^ respectively. OCT angiography (OCTA) can map the microvasculature in tissue noninvasively and has been investigated extensively in clinical ophthalmology.^16^^–^^18^

To date, most OCT devices use near-infrared (NIR) light due to its deeper penetration and the relatively easy access to commercially available light sources. However, in the past decade, using visible light for OCT illumination has drawn increasing attention due to the advent and flourish of the recently matured supercontinuum (SC) light source, which provides a smooth and powerful broadband spectrum with good spatial coherence within the visible spectral range.^19^^–^^22^ The development of vis-OCT is primarily motivated by two considerations: (1) with comparable bandwidth, shorter illumination wavelengths improve imaging resolution and (2) vis-OCT can retrieve unique tissue scattering and absorption contrasts within the visible spectral range.

The lateral and axial resolutions of OCT have linear and quadratic dependence on the center wavelength of light source, respectively. Therefore, the axial resolution is improved more by switching to visible light illumination, which provides great benefits since the major advantage of OCT over other imaging modalities is its depth resolving capabilities. The specifications of several recently reported OCT systems listed in Table 1 show that visible light illumination can provide significantly better axial resolution than NIR light with comparable bandwidth. It is also demonstrated that much smaller bandwidth is needed for vis-OCT to achieve similar resolution to NIR-OCT, which is beneficial since ultrabroad illumination bandwidth poses challenges to dispersion compensation and requires customized broadband optical components. Another advantage of vis-OCT is its sensitivity to tissue scattering and absorption in the visible spectral range. The generally higher scattering coefficients of biological tissue for visible light increase imaging contrast while sacrificing imaging depth. However, when deep penetration is not necessary, similar imaging contrast can be achieved with much lower probing power.^23^ In addition to taking advantage of different scattering properties of biological tissues at shorter wavelengths, vis-OCT can also utilize absorption information for quantitative measurement of endogenous chromophore. For example, blood vessel oximetry by vis-OCT has been investigated extensively in recent years and is one of its most important applications.^15^^,^^24^^,^^25^

**Table 1:** Summary of representative NIR- and vis-OCT systems developed recently.

Wavelength range	NIR				Visible	
Authors	Xu et al.^26^	Kolb et al.^27^	You et al.^28^	Werkmeister et al.^29^	Lichtenegger et al.^30^	Chong et al.^22^
Year	2017	2016	2015	2017	2017	2017
Axial resolution in tissue	7.5 μm	5.6 μm	1.7 μm	1.2 μm (theoretical)	0.88 μm	1.4 μm
Center wavelength	1310 nm	1000 nm	1300 nm	800 nm	555 nm	560 nm
Bandwidth	100 nm	120 nm	420 nm	170 nm	156 nm	100 nm
Sensitivity	105 dB (7 mW)	—	—	97 dB (1.5 mW)	89 dB (0.8 mW)	94 dB (0.1 mW)
Roll-off	∼0 dB/mm	—	—	10 dB/mm	24 dB/mm	5 dB/mm
A-line rate	100 kHz	1670 kHz		140 kHz	30 kHz	10 kHz
Light source	Swept source	Swept source	SC	Ti:sapphire	SC	SC
Sample	Brain (*in vivo*)	Retina	Onion	Cornea	Brain (*ex vivo*)	Retina

The first report of vis-OCT dates back to 2002,^31^ after which both the system implementation and processing algorithms have evolved significantly.^22^^,^^24^^,^^32^^,^^33^ Investigations of vis-OCT have moved from phantom demonstration^34^^–^^36^ to *in vivo* verification,^25^^,^^37^ from animal experiments^38^^,^^39^ to clinical studies,^22^^,^^40^^,^^41^ and from technical development^21^^,^^42^^,^^43^ to pathological investigations of several diseases.^44^^–^^47^ Until now, vis-OCT has been applied to imaging different anatomical sites, including the retina,^48^^,^^49^ brain cortex,^50^^–^^53^ and female reproductive tract (FRT).^23^^,^^54^ Multimodal systems combining vis-OCT with other imaging platforms have been developed to reveal comprehensive information in the examined tissue.^40^^,^^52^^,^^55^^–^^57^ In this article, we review the development of vis-OCT, including both methodology and application. We also discuss the current limitations of vis-OCT and its potential applications.

## 2 Technology Development of vis-OCT

### 2.1 Broadband Visible Light Source

OCT is based on low-coherence interferometry and requires a broadband light source with low temporal coherence but high spatial coherence. Most OCT systems use four major types of light sources: superluminescent diodes (SLDs), ultrafast lasers, SC sources, and swept sources.^2^^,^^4^ So far, SLDs have dominated in commercial OCT systems. Since SLDs resemble laser diodes except for lack of a cavity, they are relatively inexpensive compared with the other three types of light sources.^20^ The typical bandwidth of a single SLD is less than 100 nm, though the bandwidth can be broadened by different layering designs or by multiplexing.^58^ The center wavelengths of SLDs are determined by the semiconductor material and range from 670 to 1600 nm.^2^ Though state-of-the-art SLDs do not provide suitable wavelengths for vis-OCT, ultrafast lasers and SC sources can meet this requirement. Ultrafast lasers with femtosecond pulse duration provide access to high power broadband light for OCT illumination. Kerr-lens mode locked Ti:sapphire lasers belong to this group.^2^ Since Ti:sapphire lasers operate most efficiently at around 800 nm, previous investigators used barium borate (BBO) crystal to double the frequency of NIR light for vis-OCT illumination.^42^^,^^55^ However, such a visible spectrum has limited bandwidth since the efficiency for second harmonic generation of BBO crystal depends on the incident angle and cannot be optimized for the entire spectral band of a Ti:sapphire laser. The highest axial resolution achieved using this method was 12.2  μm, and the visible light spectrum covered 8 nm around 417 nm.^42^^,^^55^ To date, the majority of reported vis-OCT systems use SC sources, which achieve spectral broadening of the pump laser through nonlinear effects in a photonic crystal fiber (PCF).^19^ Swept sources are different from the first three groups of OCT light sources. Instead of illuminating the sample with light spanning a broad spectral range simultaneously, a swept source quickly sweeps through a series of continuous wavelengths. Since, ideally, the interferometer works on a single wavelength at one point in time, a single-element photodetector can replace the spectrometer to achieve spectral–domain (SD) detection of OCT signal in one sweeping cycle. Although swept sources can increase the A-line rate and effective penetration depth of an OCT system, the majority work in the NIR spectral range and no reliable visible swept source is currently available.^59^

**Fig. 1:**
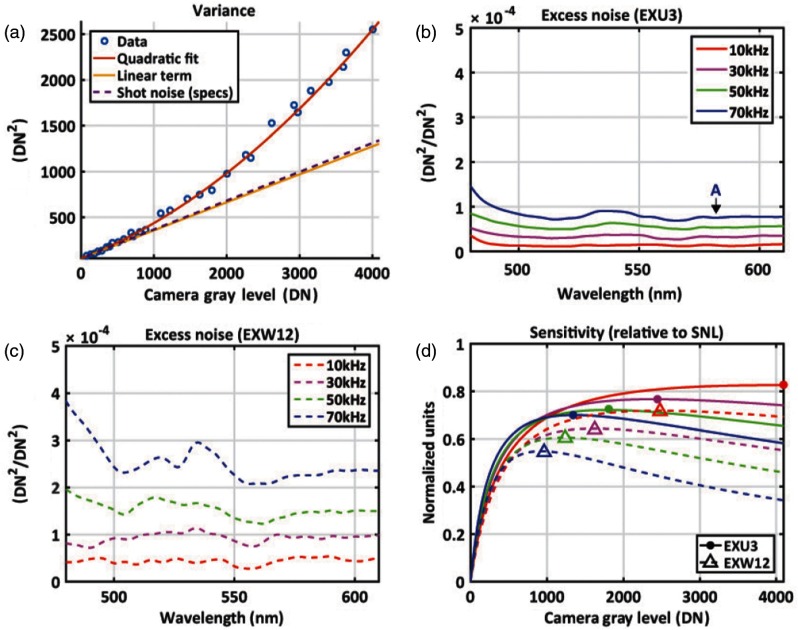
Noise characterization of SC light source for vis-OCT. Two models from NKT Photonics were compared, EXU-3 with 156-MHz PRR and EXW12 with 78-MHz PRR. (a) Quadratic fitting of the total noise variance versus camera gray level (DN), where the linear term corresponding to shot noise (solid yellow line), in agreement with the shot noise predicted from the manufacturer specified responsivity of 13,000 counts/4096 DN (dashed purple line); (b) the excess noise coefficient of the EXU-3 across visible wavelengths and a range of OCT A-line rates, which are the inverse of spectrometer exposure times; (c) the excess noise coefficient of the EXW12 is 2× larger than that of the EXU-3 at the same wavelength and A-line rate; (d) due to reduced excess noise of the higher repetition rate EXU-3 source, the camera can operate at higher gray level during imaging without introducing excess noise, enabling maximal sensitivities (filled circles) closer to the shot-noise limit (SNL), and higher than those achieved with the EXW12 source (open triangles). Reprinted with permission from Ref. 22.

In the first investigation of vis-OCT, Považay et al.^31^ built a homemade SC source by pumping PCF with a sub-15 fs Ti:sapphire laser, which provided a bandwidth of 165 nm with a 800-nm center wavelength. The spectrum was broadened by the PCF and achieved 360-nm bandwidth centered at 600 nm with a total visible power of 23 mW. A free-space time-domain (TD) OCT was built using the broadband light source, and the axial resolution reached 0.9  μm in air. Later on, with the development of PCF,^60^ commercial SC sources became increasingly popular and gradually became the top choice for vis-OCT.^25^^,^^35^^,^^61^ Commercial SC sources cover wavelengths from 400 to 2500 nm, with a total visible spectrum power beyond 1 W. The smooth broadband spectrum of SC sources provides large flexibility for illumination band selection, facilitating ultrabroadband OCT and multiband OCT.^21^ However, SC sources exhibit much higher relative intensity noise (RIN)^61^ as compared with SLDs. Therefore, the maximal sensitivity of OCT systems with SC sources is usually lower than those using SLDs, whose signal-to-noise ratio (SNR) is shot-noise limited.^20^ Since RIN of SC sources comes from the pulse to pulse amplitude variation, increasing the pulse repetition rate (PRR) can increase system sensitivity. Chong et al.^22^ reported the reduction of RIN in low-noise SC source with 156-MHz PRR rather than an SC source with 78-MHz PRR, as shown in Fig. 1. The low-noise SC source provides sufficient sensitivity for *in vivo* human eye imaging.

To date, spatially coherent light sources dominate in the development of vis-OCT. However, it should be noted that there was a successful demonstration using broadband spatially incoherent light sources, for example, xenon lamp.^34^ Since the commercial SC sources are expensive, developing low-cost alternatives within only the visible spectral range can significantly reduce the cost and promote wide adoption of vis-OCT.

### 2.2 System Implementation

Similar to NIR-OCT, which was first demonstrated in TD acquisition and later evolved into SD acquisition, vis-OCT systems also adopted SD setup after their original demonstration of concept in TD.^31^^,^^42^^,^^61^ In addition to much higher imaging speed, SD acquisition also improves sensitivity,^62^ which is important for *in vivo* functional imaging.

**Fig. 2:**
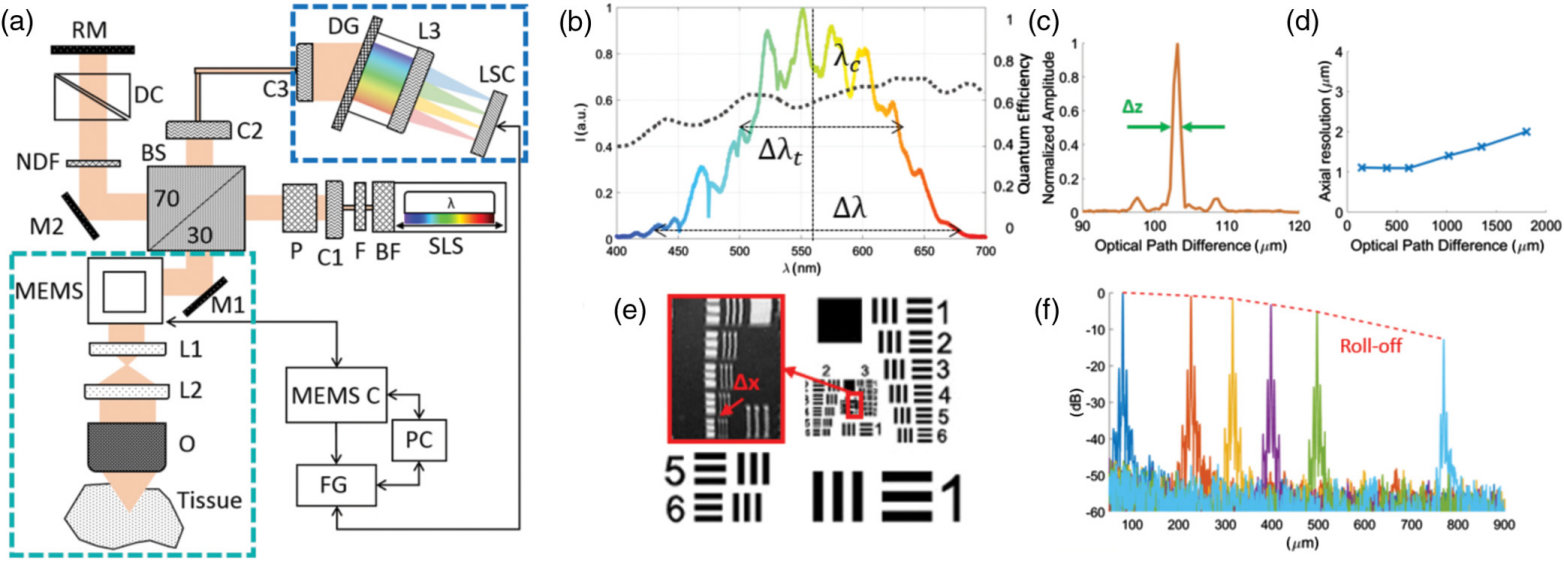
Free-space SD vis-OCT system and its characterization. (a) The schematic diagram of the experimental system. BF, bandpass filter; BS, beam splitter; C, collimator; DC, dispersion compensation; DG, diffraction grating; F, filter; FG, frame grabber; L, lens; LSC, line-scan camera; M, mirror; MEMS, microelectromechanical mirror; MEMS C, MEMS control; NDF, neutral density filter; O, objective; P, polarizer; PC, computer; RM, reference mirror; and SLS, supercontinuum light source. The blue dashed square highlights the homemade spectrometer. (b) The source spectrum measured by the homemade spectrometer, whose quantum efficiency is shown by the dotted line. The center wavelength is 555 nm and the full-width-at-half-maximum bandwidth is 156 nm. (c) The impulse response of the system showing an axial resolution of 1.2  μm in air. (d) Variation of axial resolution over the whole depth range. (e) An OCT en face image of US Air Force 1951 resolution test target shows a lateral resolution of 2  μm. (f) Measurement of system sensitivity roll-off at six depth positions. Reprinted with permission from Ref. 30.

Both free-space and fiber-based Michelson interferometers have been used in vis-OCT. Some early investigations adopted a free-space setup to avoid the excessive loss and imbalanced dispersion in the fiber couplers,^21^^,^^24^^,^^25^ while those who built line-scan SD vis-OCT could only use a free-space beam splitter and cylindrical lens to illuminate a thin slab of tissue without scanning.^15^^,^^35^ Recently, with improved performance of commercial fiber couplers in the visible spectral range, several groups switched to fiber-based setups to take advantage of the compactness, easy alignment, and easy maintenance.^22^^,^^23^ Since biological tissues have lower laser safety threshold for visible light than NIR light, vis-OCT prefers to adopt an unbalanced splitting ratio (e.g., 10/90 or 30/70) for both free-space and fiber-based Michelson interferometer to maximize the detection efficiency of the backscattered signal from the sample arm.^22^^,^^40^^,^^41^ Figure 2 shows a free-space vis-OCT system for microscopic imaging and its characterization.

There are several implementation challenges and technical limitations for vis-OCT. First, since variation in refractive indices of glasses becomes larger toward shorter wavelengths, optical lenses generally have larger chromatic aberration in the visible spectral range, which may cause extra challenges when building ultrabroadband vis-OCT. To minimize chromatic aberration, Lichtenegger et al^30^ employed reflective collimators to maintain optimal spectrometer focus across a wide spectral range extending from 450 to 650 nm. Second, the reflectivities of commonly used aluminum- and gold-coated mirrors can be significantly lower in the visible spectral range than the NIR spectral range. Therefore, if multiple mirrors are used in the sample arm of a vis-OCT system, silver coated or broadband dielectric mirrors would be preferred. Third, the A-line rate of vis-OCT is currently lower than that of NIR-OCT (Table 1), which makes it more susceptible to motion artifacts in *in vivo* application and affects its capabilities in flowmetry. This is because SLDs and swept sources are not available in the visible spectral range and longer spectrometer integration time is often used to mitigate the excess noise from the SC light source in vis-OCT. Fourth, the imaging depth of vis-OCT is limited as compared with NIR-OCT. Again, without the option for swept source, the sensitivity roll-off in vis-OCT presents a constraint to image beyond 1 mm. In addition, strong optical attenuation of biological tissue due to visible light absorption and scattering limits the illumination power for deeper tissues. Taking eye imaging as an example, while swept source NIR-OCT can image into sclera,^63^ vis-OCT can barely penetrate beyond the retinal pigment epithelium (RPE) in healthy human subjects.^40^ Therefore, vis-OCT is primarily suitable for imaging superficial tissue while providing fine resolution and different spectroscopic contrast.

### 2.3 Spectroscopic Analysis

Several groups have demonstrated functional imaging using sOCT, in which time–frequency (TF) analysis methods were used to retrieve wavelength-dependent optical properties in the samples.^64^^–^^66^ Despite the tradeoff between spectral resolution and spatial resolution, as is common with all TF analysis, sOCT can reveal the absorption and scattering spectra of biological tissues in 3-D. Therefore, the spectral features of the OCT signals can be used to identify different types of tissues and to quantify various chromophore concentrations.^67^^,^^68^

Though studies have investigated exogenous dyes to enhance the contrast of sOCT,^69^ primary interests have been focused on imaging endogenous chromophores, especially hemoglobin. The attenuation spectrum of blood, affected by both absorption and scattering, is the weighted sum of the attenuation spectra of fully oxygenated and deoxygenated blood, containing either oxyhemoglobin (${\mathrm{HbO}}_{2}$) or deoxyhemoglobin (Hb) exclusively. Therefore, spectroscopic analysis of OCT signal from blood can provide oxygen saturation (${\mathrm{sO}}_{2}$), which, combined with flow velocity and vessel diameter measurements, can ultimately provide comprehensive evaluation of oxygen metabolism.^44^^,^^50^ However, since the relative concentrations of ${\mathrm{HbO}}_{2}$ and Hb are estimated by the attenuation spectrum of blood, accurate extraction of ${\mathrm{sO}}_{2}$ requires significant attenuation spectral difference between the two types of hemoglobin within the OCT illumination spectral range. Though there were multiple trials quantifying blood ${\mathrm{sO}}_{2}$ using NIR-OCT,^70^^–^^73^ success of *in vivo* experiments was not reported until switching to visible light illumination.^15^^,^^25^ Since then the visible spectral range has become better accepted for hemodynamic study through sOCT.^24^^,^^51^

**Fig. 3:**
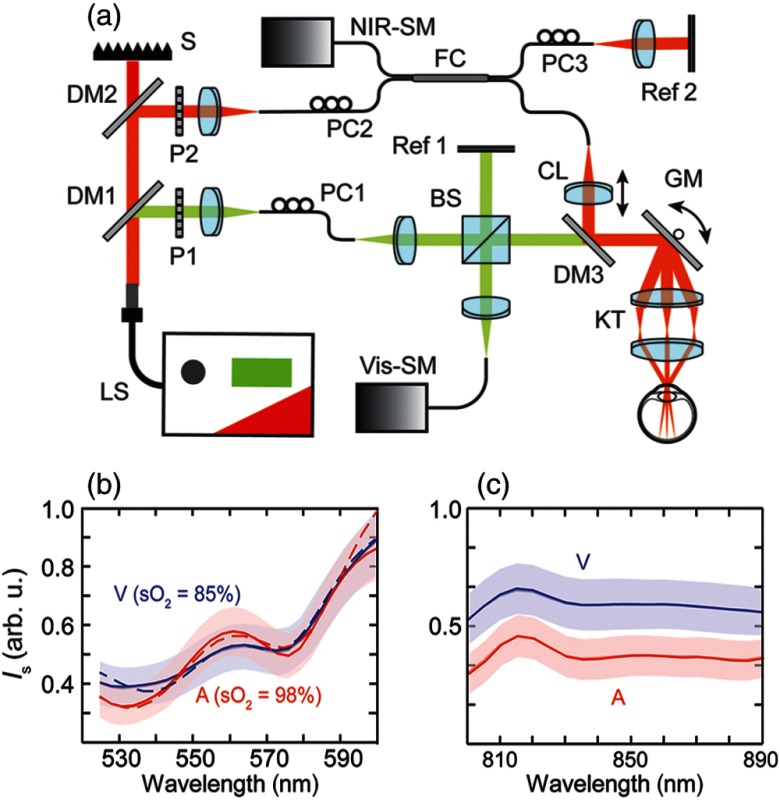
(a) The schematic diagram of a dual-band (vis and NIR) OCT system using a single SC light source for rodent retinal imaging. BS: beam splitter; CL: collimating lens; DM1 to DM3: dichroic mirrors; FC: 2×2 fiber coupler; GM: galvanometer scan mirrors; KT: Keplerian telescope; LS: supercontinuum laser source; P: polarizer; PC: polarization controller; Refs. 1 and 2: reference arms; S: beam stopper; SM: spectrometer; (b) and (c) averaged OCT intensity spectra of retinal arteries (A) and veins (V) from a mouse eye; (b) intensity spectra obtained from the visible band. Dashed lines depict fitted spectra, from which the ${\mathrm{sO}}_{2}$ values are calculated; (c) intensity spectra obtained from the NIR band. Shaded areas indicate standard error. Reprinted with permission from Ref. 21.

Quantifying ${\mathrm{sO}}_{2}$ using vis-OCT is considered more advantageous than using NIR, which has been demonstrated both theoretically and experimentally.^21^^,^^43^ First, though the attenuation spectrum is affected by both optical absorption and scattering, using the difference in the absorption spectrum is more effective in distinguishing the two types of hemoglobin in bulk blood.^74^ For example, in the wavelength range between 520 and 850 nm, the correlation coefficients between the absorption spectra and the scattering spectra of ${\mathrm{HbO}}_{2}$ and Hb are 0.948 and 0.994, respectively, indicating a much larger difference in the absorption spectra.^43^ Second, the absorption spectra of ${\mathrm{HbO}}_{2}$ and Hb in the visible spectral range are more distinctive than in the NIR spectral range. For example, there are four isosbestic wavelengths between 500 and 600 nm, while only one between 700 and 1000 nm.^73^ In addition, the absorption coefficients of hemoglobin in this visible spectral range are also two orders of magnitude higher than those in the NIR spectral range, which means that much less volume of blood is needed to sufficiently alter the spectrum of OCT signals. Third, the variations in the attenuation spectrum directly detected by OCT are more dominated by absorption in the visible spectral range. In the NIR spectral range, the absorption coefficients are one order of magnitude smaller than the reduced scattering coefficients. However, in the visible spectral range, the absorption coefficients are around twice the magnitude of the reduced scattering coefficients.^21^^,^^43^ Figure 3(a) shows a dual-band OCT system for *in vivo* retinal imaging. The averaged OCT intensity spectra from major blood vessels in a mouse eye in Figs. 3(b) and 3(c) demonstrate that the visible spectral range is a better option to distinguish blood under different oxygenation conditions. The development of vis-OCT is partly motivated by these advantages in studying oxygen-related hemodynamics. Evaluation of ${\mathrm{sO}}_{2}$ through spectroscopic analysis is one of the most important applications of vis-OCT that has been demonstrated so far.

Several spectroscopic analysis methods can be used in vis-OCT. Though these methods were proposed primarily for hemoglobin measurement and ${\mathrm{sO}}_{2}$, their applications can be extended to identify and quantify other chromophores. Note that some of these methods were proposed based on OCT systems using NIR light. However, since they can be directly applied to vis-OCT, we include them here as well.

#### 2.3.1 Spectral information extraction

There are two steps for quantifying chromophore concentration using sOCT. First, wavelength-dependent OCT signal intensities need to be extracted from the raw data. Second, these wavelength-dependent intensities are fit to the attenuation spectra of the chromophores to determine their concentrations. Extraction of spectral information is done by TF analysis.

In TD-OCT, the reference arm mirror is translated to capture backscattered light from the sample at different depths. A single-element photodetector detects the interference signal as a function z, which equals 0 when the optical path lengths from two arms match. The axial point spread function (PSF), $I_{\mathrm{TD},\mathrm{PSF}}(z)$, can be expressed as follows:

$$I_{\mathrm{TD},\mathrm{PSF}}(z)=|\Gamma (z)|\cos(2\overset{¯}{k}z),$$ (1)

where |Γ(z)| is the envelope of the autocorrelation function of the broadband light source and $\overset{¯}{k}$ is the center wavenumber. An A-line $I_{\mathrm{TD}}(z)$ can be obtained by convolving $I_{\mathrm{TD},\mathrm{PSF}}(z)$ with the backscatter profile h(z) as follows:

$$I_{\mathrm{TD}}(z)=|\Gamma (z)|\cos(2\overset{¯}{k}z)⊗h(z).$$ (2)

Spectral information can be revealed by taking the Fourier transform on both sides of the equation:

$${\overset{˜}{I}}_{\mathrm{TD}}(k)=S(k)H(k),$$ (3)

where S(k) is the measured and normalized power spectrum of the light source emission and H(k) is the sample’s spectral reflectivity.^70^^,^^75^ Here, H(k) contains spectral information from the entire A-line. To extract depth-resolved backscattered spectrum, the raw data are processed by TF methods, among which the most commonly used are wavelet transform^65^ and short-time Fourier transform (STFT).^70^^,^^73^

In FD-OCT, the broadband interference signal is detected as a function of wavelength or wavenumber. We choose a different way from above to represent the FD-OCT signal for its convenience in illustrating spectroscopic analysis. We assume that the sample consists of a series of interfaces at depth $z_{s_{1}},z_{s_{1}},\ldots$, and $z_{s_{N}}$, which, similar to the definition for TD-OCT, equals 0 when the optical path lengths on the sample and reference arms match. The reflectances of the interfaces are $R_{S_{1}},R_{S_{2}},\ldots$, and $R_{S_{N}}$, respectively. The detected interferogram $I_{\mathrm{FD}}(k)$ can be represented as

$$I_{\mathrm{FD}}(k)=S(k)\overset{N}{\underset{n=1}{\sum }}\sqrt{R_{R}R_{S_{n}}}\;\cos(2kz_{S_{n}}),$$ (4)

where S(k) is the power spectrum of the light source as in the TD-OCT and $R_{R}$ is the reflectance of the reference arm mirror. A Fourier transform converts the signal from SD to spatial domain as

$${\overset{˜}{I}}_{\mathrm{FD}}(z)=|\Gamma (z)|⊗\overset{N}{\underset{n=1}{\sum }}\sqrt{R_{R}R_{S_{n}}}\delta (\pm 2z_{S_{n}}),$$ (5)

where δ(·) is the Dirac delta function. Therefore, the depth distribution of reflective interfaces is reconstructed with |Γ(z)|, the envelope of the autocorrelation function of the light source, as the axial PSF. For biological samples, $R_{S_{n}}$ can be wavenumber dependent, affected by both the backscattering spectrum of interface $S_{n}$ and the attenuation spectrum of the tissue above it.

To detect the variation of $R_{S_{n}}$ with respect to wavenumber, two approaches were reported. The first one is to directly apply STFT to the raw interferogram $I_{\mathrm{FD}}(k)$ to obtain

$${\overset{˜}{I}}_{\mathrm{FD},k}(z,\tau_{k})=\mathrm{STFT}[I_{\mathrm{FD}}(k),w_{k}(\tau_{k},\omega_{k})],$$ (6)

where $w_{k}$ is the window function used to extract the spectrum segment of interest and $\tau_{k}$ and $\omega_{k}$ are the center wavenumber and the bandwidth of the window function, respectively. The Gaussian function is the most commonly used window function. ${\overset{˜}{I}}_{\mathrm{FD},k}(z,\tau )$ are a series of OCT A-lines calculated from different spectral subbands, with reduced axial resolution as compared with ${\tilde{I}}_{\mathrm{FD}}(z)$. Theoretically, the backscattering spectrum of any pixel along the depth profile can be obtained. However, in reality, averaging multiple adjacent points is necessary to suppress random noise.

In the second approach, STFT is applied to the reconstructed spatial domain signal, ${\overset{˜}{I}}_{\mathrm{FD}}(z)$, instead of the raw interferogram.^74^^,^^76^ We have

$$I_{\mathrm{FD},z}(k,\tau_{z})=\mathrm{STFT}[{\overset{˜}{I}}_{\mathrm{FD}}(z),w_{z}(\tau_{z},\omega_{z})],$$ (7)

where $w_{z}$ is the window function used to select the region of interest along the axial direction, and $\tau_{z}$ and $\omega_{z}$ are the center position and the width of the window function, respectively. Instead of having a number of A-lines reconstructed from different spectral segments as in the first approach, this method provides a number of spectra related to different depth ranges. However, both methods are constrained by the tradeoff between spatial and spectral resolutions.

It is common to sample a number of spectral subbands in TF analysis to recover the depth-resolved backscattered spectral intensity with finer detail. However, in certain cases, a limited number of sampling subbands can be used to simplify computation when there are no fine features in the interested spectral range. In an extreme case, the spectral information can be retrieved by calculating the ratio of OCT intensities reconstructed from only two wavelength ranges, which can either be the two subbands of a single OCT system or two OCT systems working within different spectral bands.

#### 2.3.2 Spectral fitting

Quantifying chromophore type and concentration requires the knowledge of its attenuation spectrum, which can be calculated by comparing the OCT spectra backscattered at two different depths from the same lateral position. We define the depth-resolved backscattering OCT spectrum as I(k,z) obtained using the methods described in the previous section and notate the two depths of interest as $z_{0}$ and $z_{0}+\Delta z$. Since the medium is considered homogeneous, the backscattering spectra at two depths are the same and the difference is solely caused by the attenuation of the optical path in between. According to the Beer–Lambert’s law:

$$I(k,z_{0}+\Delta z)=I(k,z_{0})e^{-2\mu_{t}(k)\Delta z},$$ (8)

where $\mu_{t}(k)$ is the attenuation spectrum of the medium primarily caused by the existence of chromophore. Since both the illumination and backscattered beams pass through the medium, there is a factor of 2 in the exponential term. $\mu_{t}(k)$ can therefore be derived as follows:

$$\mu_{t}(k)=\frac{1}{2\Delta z}\;\ln[\frac{I(k,z_{0})}{I(k,z_{0}+\Delta z)}].$$ (9)

Optical density (or absorbance) spectrum OD(k) can also be calculated; this includes both the attenuation spectrum and the optical path length:

$$\mathrm{OD}(k,\Delta z)=\frac{1}{2}\;\log_{10}[\frac{I(k,z_{0})}{I(k,z_{0}+\Delta z)}]=\log_{10}(e)\cdot \mu_{t}(k)\Delta z,$$ (10)

where the product of $\mu_{t}(k)$ and Δz is scaled by a constant since optical density is usually defined by base-ten logarithm.

The attenuation spectrum or optical density spectrum is then used to calculate the chromophore types and concentrations. We use blood ${\mathrm{sO}}_{2}$ measurement as an example to explain spectral fitting in vis-OCT. Depending on ${\mathrm{sO}}_{2}$, the attenuation coefficient spectrum of blood $\mu_{t,B}(k)$ is a weighted sum of those of fully oxygenated and deoxygenated blood and can be described as follows:

$$\mu_{t,B}(k)={\mathrm{sO}}_{2}\cdot \mu_{t,\mathrm{oxy}}(k)+(1-{\mathrm{sO}}_{2})\cdot \mu_{t,\mathrm{deoxy}}(k).$$ (11)

For either type of blood, the attenuation coefficient spectrum is a combination of absorption coefficient spectrum $\mu_{a,B}(k)$ and scattering coefficient spectrum $\mu_{s,B}(k)$:

$$\mu_{t,\mathrm{oxy}/\mathrm{deoxy}}(k)=\mu_{a,\mathrm{oxy}/\mathrm{deoxy}}(k)+W\mu_{s,\mathrm{oxy}/\mathrm{deoxy}}(k),$$ (12)

where the scattering coefficient spectrum is scaled by a packing factor W, since densely packed erythrocytes make optical scattering from each blood cell no longer independent. W relates to hematocrit of blood, H, as^77^

$$W={\left(1-H\right)}^{2}.$$ (13)

Both absorption and scattering coefficient spectra are available from the literature^77^ and the hematocrit value does not have large variation and can be measured by routine clinical practice.^78^ The attenuation coefficient spectra of fully oxygenated and deoxygenated blood can therefore be obtained. Then, the attenuation spectrum detected by OCT is compared with the two standard spectra to extract ${\mathrm{sO}}_{2}$. Although using least-squares fitting to determine the relative contribution of oxygenated and deoxygenated blood to the detected attenuation spectrum is the most common method,^24^^,^^25^^,^^41^ ${\mathrm{sO}}_{2}$ can also be directly reflected by the slope of blood attenuation spectrum within a certain wavelength range^48^ or simply by the signal attenuation ratio of two subbands.^71^^–^^73^ Yet these simplified methods can be considered special scenarios of least-squares fitting. In addition to quantifying ${\mathrm{sO}}_{2}$, vis-OCT can also measure total hemoglobin concentration by analyzing the absolute amplitude of the attenuation spectrum since the attenuation coefficient is a linear function of the chromophore concentration.^24^^,^^35^

One limitation of the current vis-OCT vascular oximetry method is that it primarily applies to the major arteries and veins, which contain enough blood volume to induce sufficient attenuation contrast within the illumination spectrum. In a single capillary, where erythrocytes often pass one at a time, there is no significant optical attenuation and the algorithms introduced above cannot easily provide reliable ${\mathrm{sO}}_{2}$ estimates. The optical properties of single capillaries more resemble that of erythrocytes than bulk blood. Though it is challenging to retrieve capillary ${\mathrm{sO}}_{2}$ from the optical attenuation spectrum, preliminary *ex vivo* studies by Liu et al.^74^ suggest that it may be possible to perform oximetry based on the backscattering spectrum of erythrocytes. However, since the backscattering spectrum of a single erythrocyte is significantly affected by its size and orientation, spatial or temporal averaging across a number of erythrocytes is required before calculating ${\mathrm{sO}}_{2}$ from spectral fitting.

## 3 Application of vis-OCT in Animal Studies

### 3.1 Retinal Imaging

Extending conventional OCT with visible light illumination, vis-OCT has been majorly applied to retinal imaging. Previous animal studies mostly focused on hemodynamic investigation of the retina while there are also reports on application of vis-OCT to rhodopsin distribution sensing and quantitative autofluorescence (AF) measurements.

#### 3.1.1 Hemodynamic investigation

Existing ophthalmic imaging tools aim at revealing the structure of retinal tissue or blood vessels. Growing evidence suggests that hemodynamic variations precede structural alterations in ocular diseases and that ${\mathrm{sO}}_{2}$ is a critical biomarker, especially in diabetic retinopathy (DR).^44^^,^^79^^,^^80^ Before vis-OCT, high resolution, noninvasive quantification of ${\mathrm{sO}}_{2}$ was measured by spectral reflectance measurements based on fundus photography or scanning laser ophthalmoscopy, which are fundamentally constrained by the lack of depth resolution and thus unable to infer absolute quantitative information.^81^

Vis-OCT enabled quantitative evaluation of ${\mathrm{sO}}_{2}$ in retinal circulation in addition to visualizing the 3-D retinal structure. The retinal tissue is nourished by both the retinal and choroidal circulations. The retinal circulation, primarily supporting the inner retina, can be characterized by the flow rates and ${\mathrm{sO}}_{2}$ of the major retinal blood vessels. While OCT measurement of blood flow rates is well-studied, it was not until 2013 that Yi et al.^25^ first demonstrated vis-OCT oximetry in major retinal arteries and veins in rodents *in vivo*. Later, it was reported that vis-OCT, as a single-imaging modality alone, can quantify a full set of metabolic parameters of retinal circulation, including total retinal oxygen delivery, oxygen extraction fraction, and metabolic rate of oxygen (${\mathrm{MRO}}_{2}$), by combining ${\mathrm{sO}}_{2}$ evaluation with blood flow velocity and vessel diameter measurement.^38^ The same study also suggested that inner retinal ${\mathrm{MRO}}_{2}$ increases during hypoxic challenge because the highly regulated inner retinal circulation needs to compensate for the deficient oxygen supply from the poorly regulated choroidal circulation during decreased systemic oxygen supply. In contrast to the retinal circulation, which has well organized and easy-to-image major blood vessels close to the inner retinal surface, the choroidal circulation is beneath the highly optically absorbing and scattering RPE, and the major arteries and veins are covered under a dense meshwork of choriocapillaris.^82^^,^^83^ Therefore, not only were the major choroidal vessels never resolved by vis-OCT but also the signals from choriocapillaris were weak due to stronger attenuation of visible light by the RPE. Though ${\mathrm{MRO}}_{2}$ of choroidal circulation has not been successfully obtained, Chen et al.^48^ reported that relative ${\mathrm{sO}}_{2}$ changes in choriocapillaris in response to systemic oxygen challenge can be revealed by vis-OCT after using speckle variance-based angiography to enhance and segment the capillaries. However, one should note that it is not a demonstration of single capillary oximetry to evaluate ${\mathrm{sO}}_{2}$ change in choriocapillaris, whose dense meshwork makes its optical properties more similar to bulk blood than single erythrocytes.

**Fig. 4:**
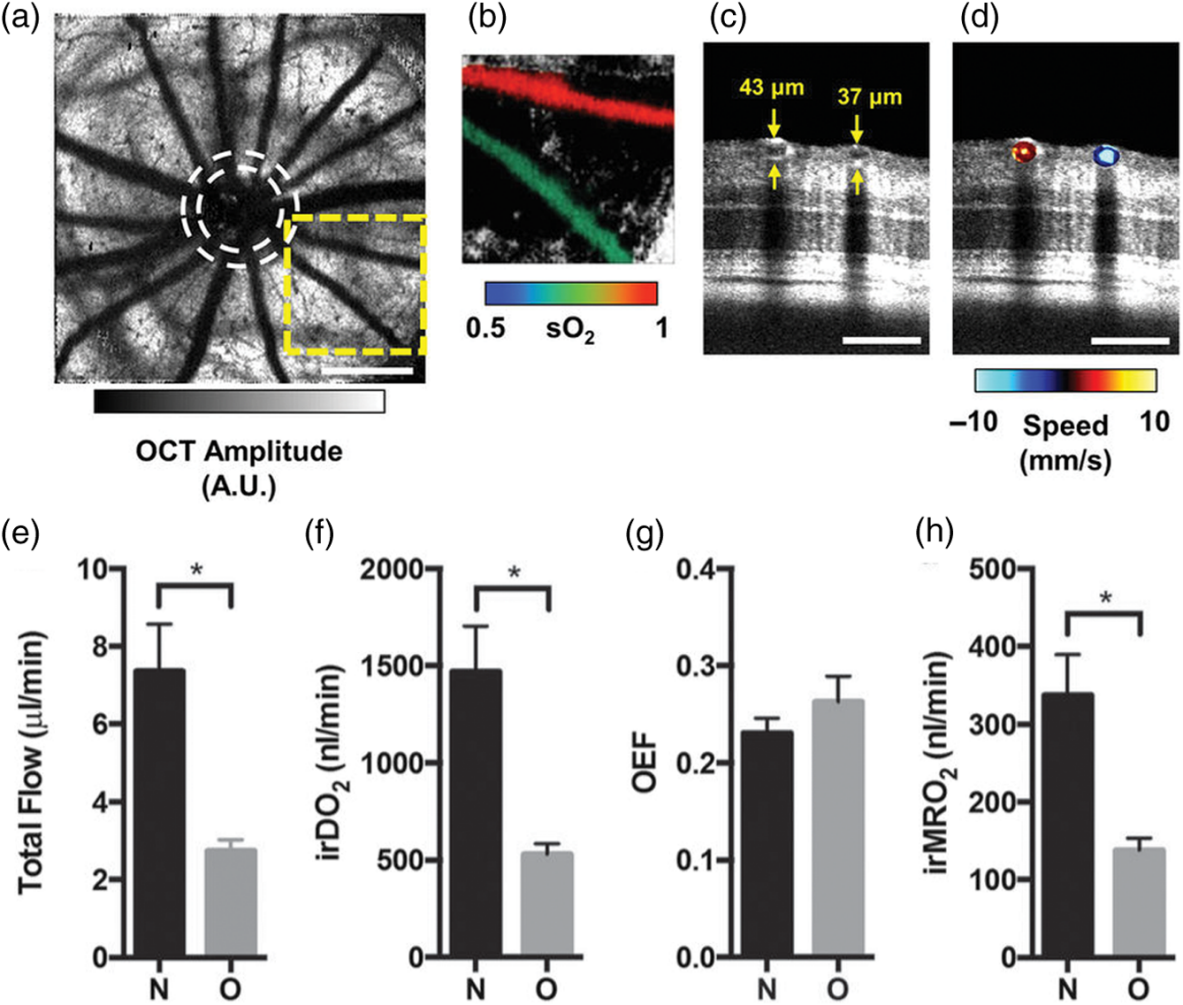
Multiparameter assessment of retinal oxygen delivery and metabolism with vis-OCT in rats. (a)–(d) Measurement of blood ${\mathrm{sO}}_{2}$, vessel diameter, and blood flow velocity from vis-OCT image. (a) En face maximum amplitude projection of the shadows of the inner retinal vessels. White dashed circles indicate the approximate inner and outer scanning paths for dual-circle Doppler OCT. A.U.: arbitrary units. Scale bar: 500  μm. (b) An artery–vein pair from the yellow dashed box in the panel (a), color-coded according to the measured ${\mathrm{sO}}_{2}$. (c) An angular section from the outer circle of the dual-circle scan, showing the artery–vein pair in the panel (b) and their respective measured diameters. Scale bar: 100  μm. (d) The same artery–vein pair color-coded according to the speed measured by Doppler OCT after phase unwrapping. Scale bar: 100  μm. (e)–(h) Hemodynamic comparison between room-air-raised controls (N, n=6) and rats with OIR (O, n=4) on postnatal day 18, (e) estimated total blood flow for N and O groups, (f) inner retinal oxygen delivery (${\mathrm{irDO}}_{2}$) measurements for N and O groups, (g) oxygen extraction fraction (OEF) measurements for N and O groups, (h) inner retinal metabolic rate of oxygen (${\mathrm{irMRO}}_{2}$) measurements for N and O groups. *p<0.05. Reprinted with permission from Ref. 47.

Vis-OCT based retinal oximetry and inner retinal ${\mathrm{MRO}}_{2}$ measurements have been applied to the investigation of hemodynamic variation on rodent models with eye diseases, including DR and retinopathy of prematurity (RoP). Liu et al.^44^ applied vis-OCT to investigate the retinal metabolic variation in genetically modified diabetic mice (Akita/+, TSP1−/−) in comparison with control mice (TSP1−/−) between the ages of 5 and 13 weeks. The study showed that, while the blood flow rate, vessel diameter, and arterial ${\mathrm{sO}}_{2}$ stay approximately constant during the observation period in both groups, the venous ${\mathrm{sO}}_{2}$ of diabetic mice gradually decreases with age, which leads to increased oxygen extraction fraction and inner retinal ${\mathrm{MRO}}_{2}$. However, both *in vivo* OCT anatomical imaging and *in vitro* histology analyses showed no vascular structural difference between the diabetic and control mice up to the end of the study, which suggested that metabolic changes may precede the structural alteration in the development of DR. In addition to investigating early biomarkers in DR, vis-OCT has also been used to study the pathophysiology of RoP in a rodent model. Soetikno et al.^47^ created 50/10 oxygen-induced retinopathy (OIR) in rat, which mimics RoP symptoms, and applied vis-OCT to study both the structural and functional evolution of retina in disease progression, as shown in Fig. 4. Compared with the control group, OIR rats showed reduced mean retinal thickness, retinal vascular density, and vascular volumetric flow rate, which primarily led to a 59% decrease in inner retinal ${\mathrm{MRO}}_{2}$ on postnatal day 18. The authors attributed the change in metabolism to decreased neuronal oxygen utilization. These studies demonstrated vis-OCT’s potential in studying inner retinal metabolism comprehensively in humans.

#### 3.1.2 Other eye imaging applications

In addition to application in hemodynamic investigation, vis-OCT has also been used for fundus AF (FAF) imaging and rhodopsin distribution sensing. In FAF imaging, the endogenous fluorophore within the retina emits a fluorescence signal upon visible light excitation. FAF signals primarily originate from the RPE, increase with RPE dysfunction, and decrease with loss of photoreceptors.^84^ Vis-OCT can be readily integrated with FAF imaging without requiring an additional light source. However, instead of using broadband light centered at 560 nm as in hemodynamic application, vis-OCT-FAF dual modality systems preferably employ light sources centered at around 480 nm, which has higher AF excitation efficiency. Since vis-OCT and FAF share the same illumination beam and scanning optics, the dual-modal system can acquire two types of images simultaneously, which are naturally coregistered.^55^^,^^56^ In addition, by taking the ratio of the vis-OCT signal intensity and AF signal intensity at RPE, true RPE AF intensity, which is independent of incident light intensity and the attenuation of eye components anterior to RPE, can potentially be quantified.^49^

The shorter illumination wavelength of vis-OCT also makes it possible to retrieve the distribution of rhodopsin, the light-sensing molecule in rod photoreceptors. The absorption peak of rhodopsin shifts from 500 to 380 nm upon photon excitation as the result of isomerization, which triggers the phototransduction cascade. Therefore, the differential image between dark-adapted and light-adapted retina taken under 500-nm illumination can highlight the existence of rhodopsin. Liu et al.^39^ demonstrated that imaging rhodopsin by vis-OCT may provide a way to functionally evaluate distribution and density of rod photoreceptors in the retina.

### 3.2 Imaging Other Anatomical Sites

In addition to the eye, vis-OCT has been used to image other anatomical sites, including mouse brain, mouse skin, and macaque FRT. However, most studies on mouse skin aimed at demonstrating technical developments of vis-OCT and its capability to measure blood oxygenation *in vivo*.^15^^,^^32^ In this section, we summarize applications of vis-OCT to brain cortex and FRT, which can either provide scientific implication or be of clinical interest.

**Fig. 5:**
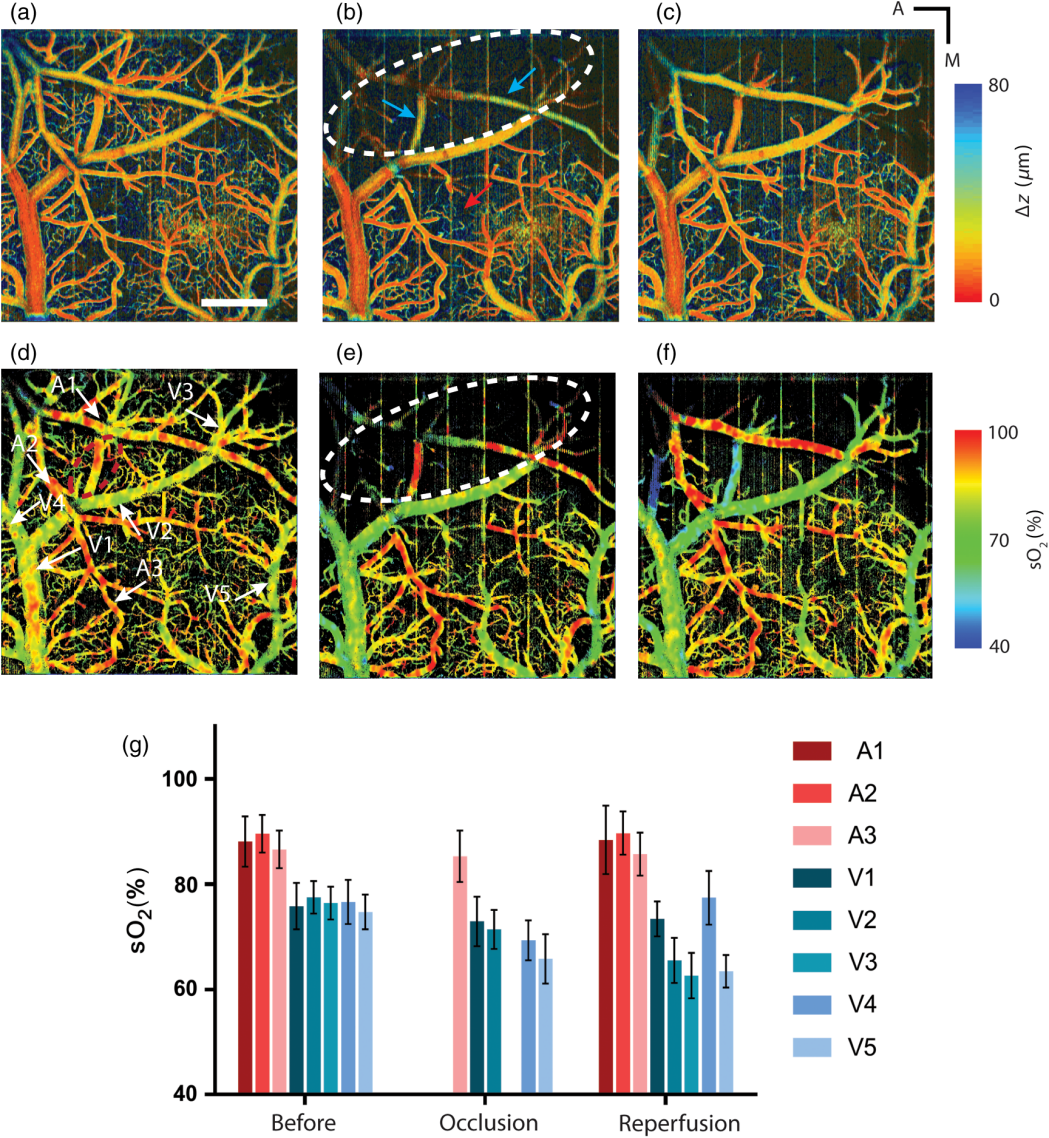
Vis-OCT study of ischemic stroke in mice. (a)–(c) Depth-coded vis-OCT angiographs in a mouse cortex. (a) Before the induction of dMCAO. Scale bar: 500  μm. (b) Immediately after the MCA occlusion. The white dashed line is the boundary of ischemic core area. The blue arrows show the reduced blood flow signal from MCA branches. The red arrow indicates newly appeared capillaries. (c) After the reperfusion of MCA branch, (d)–(f) are vascular ${\mathrm{sO}}_{2}$ map in the mouse cortex. (d) Before the induction of dMCAO. Arrows indicate the vessels where mean ${\mathrm{sO}}_{2}$ values were calculated. Dashed red line is the area with overlapped arteries and veins, (e) immediately after the MCA occlusion, (f) after the reperfusion of MCA branch, (g) mean ${\mathrm{sO}}_{2}$ values from the selected vessels. A1–A3, arteries and arterioles; V1–V5, veins and venules. Error bar, standard deviation. Reprinted with permission from Ref. 52.

Vis-OCT has been used to study hemodynamics in healthy and diseased rodent brain cortex.^50^^–^^52^ Chen et al.^51^ created a stroke model in mice by focal photothrombosis and measured morphology and ${\mathrm{sO}}_{2}$ of cerebral blood vessels by vis-OCT angiography and oximetry, respectively. Experimental results showed increased vessel diameter around the injured cortex and decreased blood ${\mathrm{sO}}_{2}$. In another study, Liu et al.^52^ created a distal middle cerebral artery occlusion (dMCAO) model in mice, which mimics human cortical stroke, by bipolar electrosurgical coagulation and used laser speckle imaging (LSI) guided vis-OCT to investigate the influence of occlusion on the brain hemodynamics, as shown in Fig. 5. Both LSI-based real-time flowmetry and OCTA showed reperfusion of middle cerebral artery (MCA) branches after dMCAO possibly due to reverse flow from communicating arterioles and anastomoses between MCA and anterior cerebral artery. Vis-OCT oximetry showed reduction in venous ${\mathrm{sO}}_{2}$ after MCA branch reperfusion while the change in arterial ${\mathrm{sO}}_{2}$ was not significant.

Colposcopy is another potential clinical application of vis-OCT. Noninvasive examination of the thickness and integrity of FRT epithelium may help to screen susceptible populations for sexually transmitted infections and to evaluate the safety of vaginal products.^23^ Vis-OCT colposcopy provides 3-D morphology of vaginal epithelium with significantly increased resolution and contrast compared with its counterpart using NIR light illumination.^54^

## 4 Human Retinal Imaging

To date, the vast majority of human retinal OCT systems use NIR light sources due to the considerations for imaging depth, patient’s comfort, and the availability of a low-cost light source and other optical components within the spectral range. With the recent development of vis-OCT and the successful demonstration of its new capabilities in *in vivo* quantitative hemoglobin mapping, a few groups have successively translated vis-OCT from tissue sample imaging and animal model studies to human eye imaging.

**Fig. 6:**
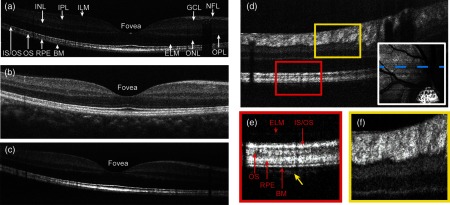
Vis-OCT imaging of human retina. (a) Vis-OCT B-scan across fovea with all anatomical structures labeled. ILM, inner-limiting membrane; NFL, neural fiber layer; GCL, ganglion cell layer; IPL, inner plexiform layer; INL, inner nuclear layer; OPL, outer plexiform layer; ONL, outer nuclear layer; IS/OS, inner/outer segment junction; OS, outer segment of photoreceptor; RPE, retinal pigmented epithelium; and BM, Bruch’s membrane; (b) B-scan taken by a commercial NIR-OCT system at the same location; (c) averaged vis-OCT image from eight consecutive B-scans. The motion artifact was removed by aligning the adjacent B-scans; (d) vis-OCT B-scan around ONH. The inset at the bottom-right corner shows the en face vis-OCT image, from which the B-scan is taken; (e)–(f) magnified images from the squared areas in the panel (d). The yellow arrow points to the signal from the choriocapillaris immediately beneath BM. The anatomical structures in the outer retina are labeled in the panel (e). Reprinted with permission from Ref. 40.

Yi et al.^40^ reported the first vis-OCT system for human retinal imaging. The system, built on an optical table, used wavelengths between 496 and 632 nm for illumination, which was obtained by bandpass filtering the output of a commercial SC light source (SuperK EXW-6, NKT Photonics). The interferometer adopted a free-space setup using 70/30 beam splitter, with 70% of the reflected sample arm energy delivered to SD detection. The system’s sensitivity was 86 dB with 226-μW illumination and 37-μs exposure time. In addition to vis-OCT imaging, the system could switch to scanning laser ophthalmoscope (SLO) mode, which shared the same illumination beam, scanning mirrors, and relaying optics as vis-OCT. SLO en face images updated at a speed of 6 frames/s, which facilitated identification of region of interest and focus adjustment before vis-OCT imaging. The vis-OCT images were taken with 10-by-10-degree field-of-view centered on either fovea or optic nerve head and were compared with NIR-OCT images acquired by a commercial machine, as shown in Fig. 6. Both vis-OCT and NIR-OCT exhibited similar anatomical structures of the retina in B-scans. However, vis-OCT showed much increased contrast of retinal nerve fiber layer (RNFL) and the layers in the outer retina, including the boundary between the photoreceptor inner and outer segments (IS/OS), the OS of photoreceptors, RPE layer, and Bruch’s membrane (BM).

**Fig. 7:**
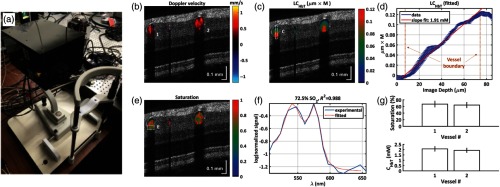
Vis-OCT ophthalmoscope for human retinal imaging. (a) A photograph showing the sample arm (covered) mounted on the ophthalmoscope platform; (b) image of measured Doppler velocities overlaid on structural OCT B-scan; (c) cumulative hemoglobin in retinal vessels exhibits a characteristic downward “crescent” shape, due to a larger cumulative path length at the distal end of the vessel; (d) the hemoglobin concentration in the marked vein was estimated to be 1.91 mM, corresponding to 12.3  g/dL; (e) oxygen saturation mapping in retinal vessels; (f) spectroscopic fit for the distal portion of the vein indicated by the dashed square in panel e; (g) the means and standard deviations of ${\mathrm{sO}}_{2}$ and total hemoglobin concentration ($C_{\text{HbT}}$) for vessels 1 and 2 (labeled in b), which are calculated from a series of continuous measurements within a period of 3 s. The measured saturations for the two vessels are 67.2±8.8% and 64.4±8.2%, respectively. The measured $C_{\text{HbT}}$ values for the same vessels are 2.08±0.22  mM (13.4±1.4  g/dL) and 1.94±0.20  mM (12.5±1.3  g/dL), respectively. Imaging was performed with 10 kHz A-line rate and 100  μW power on pupil. Reprinted with permission from Ref. 22.

After the first demonstration of vis-OCT in humans, different groups have been working on optimizing both the system hardware and processing algorithms toward clinical application, especially ${\mathrm{sO}}_{2}$ mapping. Chong et al.^22^ developed a fiber-based vis-OCT ophthalmoscope, whose appearance resembled commercially available clinical systems, as shown in Fig. 7. Compared with previous systems, several improvements were made to increase imaging sensitivity. The upgraded SC light source (SuperK EXU-3, NKT Photonics) had a PRR of 156 MHz, twice that of its predecessor, which suppressed the noise by more than 50%. The splitting ratio of the fiber-based interferometer also increased from 70/30 to 90/10, which allowed higher detection efficiency for backscattered signal from the sample arm. The improved system had 96-dB sensitivity with 150-μW illumination power at cornea and 98-μs exposure time. The *in vivo* axial resolution was quantified to be less than 2  μm by measuring the FWHM of the inner-limiting membrane. Split-spectrum analysis of the retinal structure image showed that inner retinal layers produced a stronger signal at shorter wavelengths, whereas the RPE and BM layers produced a stronger signal at longer wavelengths. Functional information, including blood flow velocity, total hemoglobin concentration, and ${\mathrm{sO}}_{2}$, were measured by Doppler and sOCT, respectively.

Unlike NIR-OCT, which has already been adopted in clinical environments for over a decade, vis-OCT has just been translated to human eye imaging and more studies need to be done regarding hazard of visible light illumination on the retina. Exposure to a spatially coherent light source in medical imaging can be hazardous to retina through thermal, thermoacoustic, and photochemical effects.^85^ Human retina is more susceptible to damage from visible light due to the sensitivity of photoreceptors to the visible range of electromagnetic spectrum and the higher energy of light with shorter wavelengths. Recently, concerns have been raised against blue light (400 to 490 nm), which is experimentally demonstrated to induce damage to RPE and photoreceptors not only upon high intensity acute exposure but also with long-term subthreshold exposure.^86^ To minimize the hazard associated with visible light, investigators always select the illumination band of vis-OCT beyond 500 nm and strictly follow the latest ANSI Z136.1–2014 American National Standard for Safe Use of Lasers published by Laser Institute of America to calculate the maximum permissible exposure (MPE) for retinal imaging.^87^ The illumination power of vis-OCT is currently limited within 10% of MPE to ensure the eye safety and visual comfort of the imaged human subject.^22^^,^^40^

Currently, the quality of vis-OCT retinal imaging is primarily limited by two factors. First, much lower absolute illumination power is used for vis-OCTs than commercial NIR-OCTs. The maximum reported power for vis-OCT human eye imaging is 226  μW, around one fifth of commercial NIR-OCT, which uses around 1 mW.^40^ Second, the SC light sources in vis-OCT systems have stronger inherent noise than SLDs in NIR-OCT systems. Therefore, lower illumination power and higher light source noise in vis-OCT collectively lead to relatively lower SNR than commercial NIR-OCT. Chen et al.^41^ developed a statistical fitting-based method to retrieve unbiased estimation of vis-OCT signal intensity with moderate SNR for human retinal oximetry. According to Chen et al., the noise-affected OCT signal intensity follows the nonsymmetrical Rice distribution. Therefore, directly applying spatial averaging for noise suppression results in biased estimation, which cannot be ignored if the SNR is lower than 20 dB. Chen et al. showed that statistical fitting using Rice distribution of OCT signals in retinal blood vessels reduced ${\mathrm{sO}}_{2}$ measurement uncertainty by nearly one half. The study also demonstrated retinal oximetry in healthy volunteers with different image SNRs. The arterial ${\mathrm{sO}}_{2}$ was consistently higher than 90% while the venous ${\mathrm{sO}}_{2}$ was lower than 80%. Statistically significant differences were also found in major retinal artery and vein branches.

## 5 Perspective of vis-OCT

Vis-OCT has gradually gained attention from the research community, especially during the past 5 years, in part due to the availability and improvement of commercial SC light sources. The major difference between vis-OCT and the commonly used NIR-OCT is the shorter illumination wavelength. All the technical developments for NIR-OCT can potentially be applied to vis-OCT as well, including flowmetry based on Doppler phase variation, angiography based on motion contrast, spectroscopic analysis, polarization-sensitive detection, and etc. Vis-OCT is attractive to investigators, who need higher axial resolution and spectral contrast. On the other hand, the shorter wavelengths also compromise imaging penetration depth into biological tissues and lead to discomfort for eye imaging. However, we believe that vis-OCT is a useful technology whose potential in biological imaging is yet to be fully explored.

Vis-OCT imaging can benefit from several technical improvements. Since the bottleneck for SNR is the excess RIN from SC light source, a low-noise broadband light source can further increase image quality, especially for applications, such as human eye imaging, where illumination power must be strictly limited. Indeed, vis-OCT does not need the ultrawide emission band from an SC laser. A low-cost, bandlimited source between 500 and 600 nm with minimal RIN is the desired light source for vis-OCT. Random speckle pattern is another source of noise. Although vis-OCT is relatively less affected by speckle noise than NIR-OCT due to finer resolution, it can still degrade image quality and prevent reliable chromophore quantification. The traditional method to reduce speckle noise is to average multiple sequentially acquired images at the same location, which works for flowing blood, though it is not very effective for static tissue.^24^^,^^72^ Recently, Liba et al.^88^ developed speckle-modulating OCT, which used a moving diffuser to induce time-varying speckle patterns for static tissue so that they can be removed by averaging temporarily. This speckle reduction method improved the effective resolution of NIR-OCT and may be valuable for vis-OCT when high *in vivo* resolution is required and motion is not a significant concern.

We expect that vis-OCT will play an increasingly important role in biomedical imaging in the near future. Eye imaging will still be a major application of vis-OCT. Development and optimization of a portable vis-OCT ophthalmoscope will facilitate routine clinical imaging on both healthy and diseased subjects. It is of great interest to study inner retinal oxygenation of patients with DR and retinal occlusive diseases.^89^ In addition to retinal hemodynamic investigations, vis-OCT may also be applied to early detection of glaucoma and age-related macular degeneration (AMD). It is believed that, in glaucoma, morphological changes in RNFL precede severe disease progression. However, standard OCT has limited ability to detect abnormalities of the retinal microstructure.^90^ Vis-OCT will be likely to bring more benefit in clinical diagnosis and management of glaucoma since it has demonstrated higher contrast and higher resolution in imaging RNFL.^22^^,^^40^ In addition, before changes in retinal layer thickness can be observed, subdiffraction cellular damages were found to be encoded in the scattering spectrum within the visible spectral range,^45^^,^^91^^,^^92^ which can potentially be detected by vis-OCT. AMD is another leading cause of blindness whose early diagnosis draws attention from both researchers and clinical practitioners. Aging of the RPE and loss of the RPE melanin play important roles in pathogenesis of AMD. Clearly resolving this 10-μm-thick cell monolayer is necessary before it can be thoroughly studied by OCT. Currently, commercial NIR-OCT has limited axial resolution (5 to 7  μm) to accurately characterize the 10-μm-thick monolayer of pigmented cell.^93^ Vis-OCT, with higher than 2-μm *in vivo* resolution, has proven to be effective in imaging RPE with greater clarity.^40^^,^^93^ In addition, since the RPE melanin has stronger absorption and scattering within the visible spectral range than within NIR spectral range,^94^ vis-OCT images can be more sensitive to the variation in the RPE melanin concentration. Therefore, it might be of interest to apply vis-OCT to investigate the RPE melanin in AMD. Previous studies suggest estimating the melanin concentration in the RPE by quantitatively measuring the optical absorption of RPE.^94^^,^^95^ However, OCT imaging is based on backscattering and the absorption measurement requires comparing the signal intensities of reference tissue layers above and beneath the RPE. Vis-OCT, due to strong attenuation of illumination and backscattered light within the RPE, cannot effectively image the underlying ocular tissues.^40^ Since it is challenging for vis-OCT to directly measure optical absorption of the RPE, it might be more effective to quantify the melanin concentration based on the appearance of RPE in high resolution vis-OCT images. Wilk et al.^96^ demonstrated qualitatively that the melanin concentration of the RPE may be reflected by its intensity and thickness in the OCT image. Therefore, with further numerical and experimental studies, a quantitative relationship might be obtained to facilitate studying melanin concentration using vis-OCT.

In addition to ophthalmology, we may also expect that vis-OCT will become an important research tool for brain imaging. Hemodynamic investigation of healthy and diseased animal models will continue to provide insight into brain metabolism under different conditions. Combining vis-OCT with a chronic transparent cranial window makes it possible to monitor cortex vasculature functions longitudinally. Application of vis-OCT may also go beyond vascular imaging. A recent study by Lichtenegger et al.^30^ suggested that vis-OCT can provide spectroscopic contrast of *in vitro* brain tissue, which helps distinguish gray matter, white matter, and, most importantly, neuritic amyloid-beta plaques, a feature of Alzheimer’s disease.

In summary, vis-OCT is an exciting functional OCT extension, which has evolved rapidly in the past few years. Compared with its counterpart using NIR light, vis-OCT has higher axial resolution and can provide additional imaging contrast for spectroscopic analysis while sacrificing imaging depth, maximum achievable sensitivity, and highest possible imaging speed due to either fundamental or existing technical limitations. Therefore, vis-OCT should be viewed as providing complementary structural and functional information to NIR-OCT. Vis-OCT has been and will be continuously applied to various studies, among which the most important ones are likely to be in ophthalmology and brain imaging. Future technical improvements regarding the light source, system implementation, and processing algorithms will further broaden the application of vis-OCT in both fundamental investigations and clinical practices.
